# NcRNAs: a potential treatment for spinal cord injury

**DOI:** 10.3389/fncel.2025.1645639

**Published:** 2025-09-01

**Authors:** Jie Bao, Wenhui Zhi, Sheng Qi, Haolong Mo, Ruzhuan Liu, Chunhui Guo

**Affiliations:** 1 Graduate School, Guangxi University of Chinese Medicine, Nanning, Guangxi, China; 2 Ruikang Hospital Affiliated to Guangxi University of Chinese Medicine, Nanning, Guangxi, China

**Keywords:** spinal cord injury, SCI, circular RNA, long non-coding RNA, microRNA

## Abstract

Spinal cord injury (SCI) is a serious disorder that affects sensory, motor, and autonomic functions. Its pathological process is divided into two stages: primary and secondary injury. The secondary injury involves a variety of biological cascade reactions, leading to an imbalance in the spinal cord microenvironment. Non-coding RNAs (ncRNAs) play a crucial regulatory role in the pathophysiological process of spinal cord injury, including long non-coding RNAs (lncRNAs), circular RNAs (circRNAs), and microRNAs (miRNAs), all of which are involved in processes such as axonal regeneration, oxidative stress, inflammatory response, autophagy, and apoptosis. Although the pathophysiological process of spinal cord injury has been partially elucidated, its pathogenesis is not yet fully understood, and effective treatments are limited. This article reviews the regulatory role and molecular mechanisms of ncRNAs in the development and progression of spinal cord injury and proposes strategies for treating spinal cord injury by regulating ncRNAs.

## 1 Introduction

Spinal cord injury (SCI) is one of the most complex disorder, with pathological consequences that affect sensory, motor, and/or autonomic functions (Costăchescu et al., 2022; Li et al., 2020). The primary reason for this is that the spinal cord is the main communication system between the brain and the body, ensuring the exchange of information and signals for coordinated activities (Costăchescu et al., 2022). Its injury can lead to interruptions in neural circuits and connections, resulting in neural dysfunction (Yuan et al., 2021). Specifically, spinal cord injury can lead to damage to blood flow, respiration, body temperature, body pressure, and sensation, as well as permanent consequences such as paralysis, autonomic dysfunction, and neuropathic pain (Costăchescu et al., 2022).

The pathological process of spinal cord injury is divided into two stages. The primary injury is the first stage, which includes the death of neurons and glial cells, bleeding, foreign body invasion, and disruption of the axonal network (Ahuja et al., 2020). The second stage is secondary injury, which can last for several weeks and involves a series of biological cascade reactions, such as neuroexcitotoxicity, vascular dysfunction, inflammatory damage, apoptosis, free radical production, and lipid peroxidation (Ahuja et al., 2020; Anjum et al., 2020). At the same time, these factors significantly contribute to the imbalance of the spinal cord microenvironment. However, our understanding of the spinal cord microenvironment after spinal cord injury remains very limited (Seblani et al., 2023; Ortega et al., 2023; Chio et al., 2021; Peng et al., 2024). The “microenvironmental imbalance” after spinal cord injury is defined as the loss of homeostatic balance in tissues, cells, and molecules at different times and locations, which exacerbates and accelerates the progression of spinal cord injury (Peng et al., 2024; Fan et al., 2018). Studies have found that this process may involve abnormal gene expression, such as ncRNAs playing an important regulatory role in the pathophysiology of spinal cord injury.

Researchers have discovered that various non-coding RNAs (ncRNAs), such as long non-coding RNAs (lncRNAs), circular RNAs (circRNAs), and microRNAs (miRNAs), exhibit differential expression following central nervous system (CNS) injuries,such as spinal cord injury (Li et al., 2021). Furthermore, lncRNAs and circRNAs can function as competing endogenous RNAs (ceRNAs) to sponge and inhibit the expression of miRNAs, thereby creating complex regulatory networks (Guo et al., 2025). Existing studies have demonstrated that ncRNAs, including circRNAs (Xu et al., 2021; Yuan et al., 2020), lncRNAs (Zhou and Yu, 2021; Cai et al., 2023), and miRNAs (Baichurina et al., 2021; Xu et al., 2024), are involved in the pathophysiological processes of spinal cord injury, such as axonal regeneration, oxidative stress, inflammatory responses, autophagy, and apoptosis.

Although basic research has clarified the pathophysiological processes of spinal cord injury, the underlying pathogenic mechanisms remain incompletely understood, and effective treatment options are still limited. Therefore, this article aims to review and categorize the regulatory roles and molecular mechanisms of ncRNAs in the development and progression of spinal cord injury and proposes strategies for treating spinal cord injury by targeting the pathogenic mechanisms of ncRNAs.

## 2 NcRNA and spinal cord injury

ncRNAs refer to RNA molecules that do not have the potential to encode proteins, making up the vast majority of RNAs and accounting for approximately 98–99% of the RNA produced by the mammalian genome (Arraiano, 2021; Sun et al., 2022). This category includes RNAs with specific functions, such as rRNA, tRNA, snRNA, snoRNA, and miRNA. Additionally, lncRNA and circRNA are new members of the non-coding RNA family that can act as sponges for miRNAs, thereby reducing their expression levels (Yao X, et al., 2024). However, increasing evidence indicates that ncRNAs play a crucial role in spinal cord injury, suggesting their significant potential in the diagnosis, evaluation, and treatment of spinal cord injury.

MiRNAs are highly conserved single-stranded ncRNAs typically composed of 20–22 nucleotides (Yao X, et al., 2024). The typical function of miRNAs is to negatively regulate gene expression by binding to target mRNAs, leading to mRNA degradation or inhibition of translation (Yao X, et al., 2024). Studies have shown that each miRNA can target hundreds of genes and can regulate more than one-third of human genes (Sun et al., 2022), playing a role in the regulation of neurological disorders and disorders associated with nerve trauma (Arzhanov et al., 2022; Silvestro and Mazzon, 2022). For example, miR-7b-3p plays a dual role in supporting cortical plasticity and neuroprotection after spinal cord injury (Ghibaudi et al., 2021). The overexpression of miR-423-5p can act as a polarizing regulator of microglia, inhibiting the polarization of the M1 phenotype by suppressing the expression of NLRP3 (NOD-like receptor family pyrin domain-containing 3), and can be used for the treatment of spinal cord injury (Cheng et al., 2021).

LncRNAs are a class of RNA transcripts longer than 200 nucleotides that, despite lacking the ability to encode proteins, resemble mRNA (Salvatori et al., 2020). They possess various epigenetic regulatory forms, including DNA methylation, histone modification, and regulation of miRNAs (Yao X, et al., 2024). Additionally, numerous studies indicate that lncRNAs play significant roles in development, metabolism, as well as in the function of the nervous and immune systems (Chen and Kim, 2024). For instance, lncAirsci is significantly upregulated during the acute inflammatory phase of spinal cord injury. However, the inhibition of lncAirsci can alleviate the inflammatory response through NF-κB (Nuclear factor-κB) signaling pathway, promoting functional recovery (Zhang T, et al., 2021).

CircRNAs are generated from precursor mRNA through back-splicing of exons and are widely expressed in tissue-specific and developmental stage-specific patterns (Yao X, et al., 2024). Increasing evidence suggests that circRNAs regulate various cellular processes by acting as miRNA sponges, anchors for cRBPs (circRNA-binding proteins), transcriptional regulators, molecular scaffolds, and sources for the translation of small proteins/peptides (Misir et al., 2022). Unlike linear RNAs, circRNAs are circular molecules with covalently closed loop structures and are involved in a wide range of biological processes. Disruptions in their expression can lead to cellular dysfunction and disorder (Chen, 2016). A substantial body of evidence indicates that circRNAs are highly expressed in the spinal cord and play crucial roles in multiple processes of neurological disorders. For example, CircHIPK3 mitigates inflammation and neuronal apoptosis after spinal cord injury by regulating the miR-382-5p/DUSP1 (Dual-specificity phosphatase 1) axis (Yin et al., 2022). CircCDR1 as regulates scar formation, inflammation, and neural regeneration after spinal cord injury through the miR-7a-5p/TGF-*β* (Transforming growth factor-β) R2 axis (Wang et al., 2024).

## 3 NcRNA regulation of synaptic function after spinal cord injury

**Figure 1:**
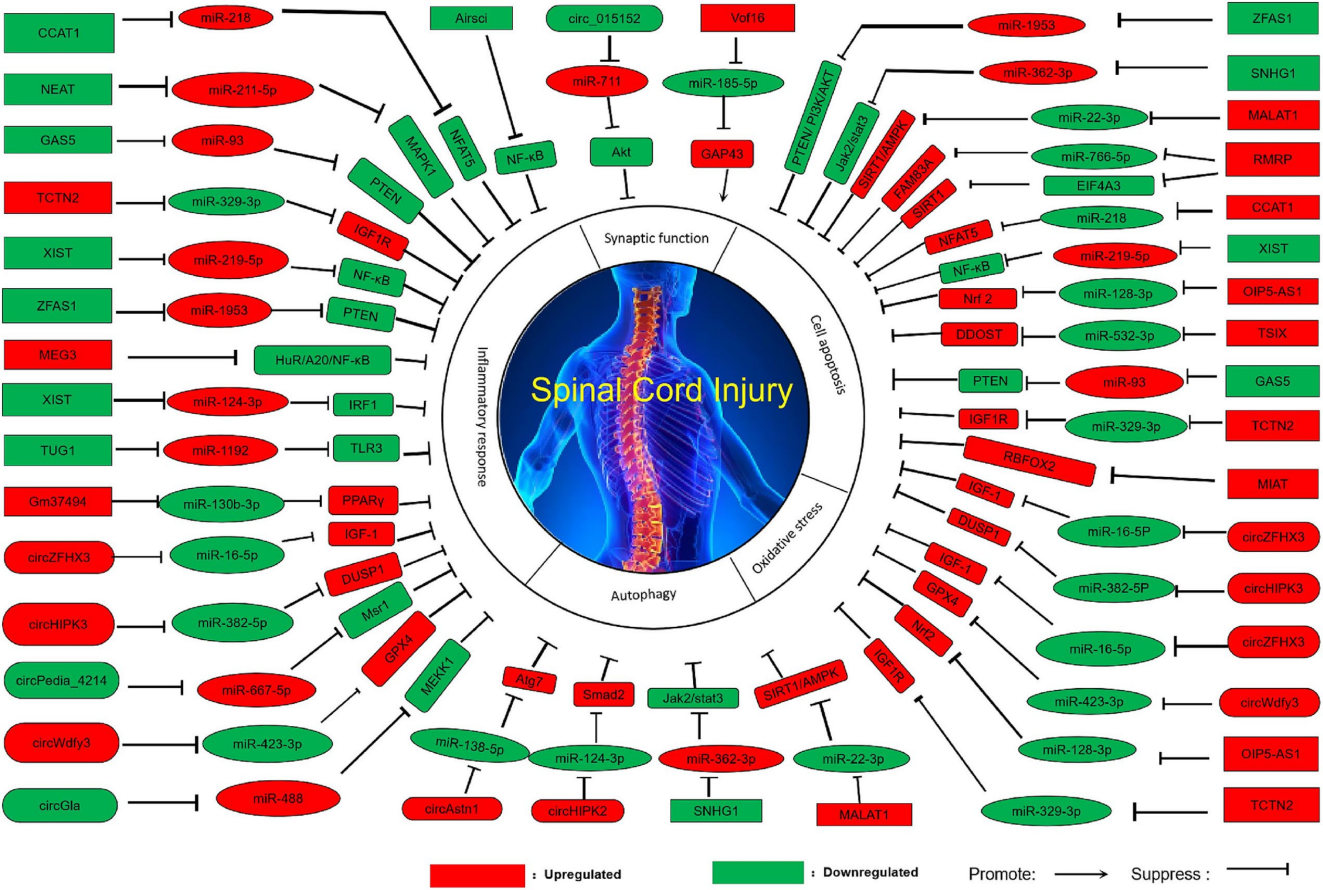
Pathologic modulation of spinal cord injury by ncRNAs.

After spinal cord injury, inadequate axonal regeneration often leads to poor recovery, which is one of the most pressing challenges in the treatment of spinal cord injury (Bie et al., 2021). Developing successful regenerative strategies to reconnect axons within the central nervous system is crucial for spinal cord injury research (Shi et al., 2024). A large body of research results indicate that the biological functions of ncRNA are related to synaptic function (Table 1; Figure 1).

**Table 1:** Regulation of the pathogenesis of spinal cord injury by ncRNAs.

ncRNAs	Expression	RNA regulatory axis expression	SCI pathogenesis	References
NcRNAs regulation of synaptic function after spinal cord injury				
circ_015152	↓	Circ_015152↓/ miR-711↑/ Akt↓	synaptic function ↓	Liu et al. (2020)
lncVof16	↑	lncVof16↑/miR-185-5p↓/GAP43↑	synaptic function ↑	Hu et al. (2024)
miR-132 miR-222 miR-431	↑	miR-132↑ miR-222↑ miR-431↑	synaptic function ↑	Zhang N, et al. (2021)
let-7 b-5p	↑	let-7 b-5p↑/LRIG3↓	synaptic function ↑	Liu et al. (2024)
NcRNA regulation of oxidative stress after spinal cord injury				
circZFHX3	↑	circZFHX3↑/ miR-16-5p↓/IGF-1↑	oxidative stress ↓	Tian et al. (2022)
circWdfy3	↑	circWdfy3↑/ miR-423-3p↓/GPX4↑	oxidative stress ↓	Shao et al. (2024a)
lncOIP5-AS1	↑	lncOIP5-AS1↑/miR-128-3p↓/Nrf2↑	oxidative stress ↓	Jiang et al. (2024)
lncTCTN2	↑	lncTCTN2↑/miR-329-3p↓/IGF1R↑	oxidative stress ↓	Liu et al. (2022)
miR-340-5p	↑	miR-340-5p↑/P38/MAPK↓	oxidative stress ↓	Qian et al. (2020)
NcRNA regulation of inflammatory response after spinal cord injury				
circZFHX3	↑	circZFHX3↑/ miR-16-5p↓/IGF-1↑	Inflammatory ↓	Tian et al. (2022)
circHIPK3	↑	circHIPK3↑/miR-382-5p↓/DUSP1↑	inflammatory ↓	Yin et al. (2022)
circPedia_4,214	↓	circPedia_4,214↓/miR-667-5p↑/Msr1↓	inflammatory ↓	Cao et al. (2023)
circWdfy3	↑	circWdfy3↑/ miR-423-3p↓/GPX4↑	inflammatory ↓	Shao et al. (2024a)
circGla	↓	circGla↓/ miR-488↑/MEKK1↓	inflammatory ↓	Shao et al. (2024b)
lncAirsci	↓	lncAirsci↓/ NF-κB↓	inflammatory ↓	Zhang T, et al. (2021)
lncCCAT1	↓	lncCCAT1↓/miR-218↑/NFAT5↓	inflammatory ↓	Xia et al. (2020)
lncNEAT	↓	lncNEAT↓/ miR-211-5p↑/MAPK1↓	inflammatory ↓	An et al. (2021)
lncGAS5	↓	lncGAS5↓/ miR-93↑/PTEN↓	inflammatory ↓	Cao et al. (2021)
lncTCTN2	↑	lncTCTN2↑/ miR-329-3p↓/IGF1R↑	inflammatory ↓	Liu et al. (2022)
lncXIST	↓	lncXIST↓/ miR-219-5p↑/NF-κB↓	inflammatory ↓	Zhong et al. (2021)
lncZFAS1	↓	lncZFAS1↓/ miR-1953↑/ PTEN↓	inflammatory ↓	Chen et al. (2021)
lncMEG3	↑	lncMEG3↑/HuR/A20/NF-κB↓	inflammatory ↓	Zhou et al. (2022)
lncXIST	↓	lncXIST↓/ miR-124-3p↑/IRF1↓	inflammatory ↓	Yang et al. (2023b)
lncTUG1	↓	lncTUG1↓/ miR-1192↑/TLR3↓	inflammatory ↓	Ju et al. (2023)
lncGm37494	↑	lncGm37494↑/ miR-130b-3p↓/PPARγ↑	inflammatory ↓	Shao et al. (2020)
let-7b-5p	↑	let-7b-5p↑/ LRIG3↓	inflammatory ↓	Liu et al. (2024)
miR-340-5p	↑	miR-340-5p↑/ P38/MAPK↓	inflammatory ↓	Qian et al. (2020)
miR-124-3p	↑	miR-124-3p↑/ MYH 9↓/PI3K/AKT↑/NF-κB↓	inflammatory ↓	Jiang et al. (2020)

Circ_015152 can act as a sponge for miR-711. When its expression is reduced, the expression of miR-711 increases, inhibiting the activation of the Akt (Protein kinase B) pathway, thereby promoting axonal damage in the spinal cord (Liu et al., 2020). High expression of lncVof16 reduces the expression level of miR-185-5p through the miR-185-5p/GAP43 (Growth-associated protein 43) axis, thereby indirectly increasing the expression of GAP43, enhancing self-repair and promoting axonal growth to improve the prognosis after spinal cord injury (Hu et al., 2024). Overexpression of miR-132, miR-222, and miR-431 can significantly enhance axonal regeneration and functional recovery (Zhang N, et al., 2021).

Overexpression of let-7b-5p maintains the integrity of myelin by inhibiting its downstream target gene LRIG3 (Immunoglobulin domain-containing protein 3), and promotes axonal growth, ultimately restoring the functional ability of spinal cord injury mice (Liu et al., 2024). Perhaps by regulating the expression of ncRNAs, we can improve axonal regeneration function and thus achieve recovery and improvement of motor function.

Extensive studies have demonstrated that exosomes serve as critical intercellular communication tools for transferring ncRNAs between neurons and bodily fluids (Wang et al., 2022). Additionally, they possess the advantage of acting as drug delivery vehicles that can transport therapeutic agents to recipient cells without activating the immune system (Guo et al., 2025), suggesting that the future combination of exosomes and ncRNAs may become a key strategy for improving spinal cord injury treatment. Furthermore, different ncRNAs exhibit distinct functional roles, and future research might focus on coordinated multi-target regulation to achieve enhanced synaptic function repair, thereby optimizing therapeutic outcomes.

## 4 NcRNA regulates oxidative stress after spinal cord injury

Many experimental and clinical studies have found the key role of ROS (Reactive oxygen species) and lipid peroxidation in the development of spinal cord injury (Chio et al., 2022; Hou et al., 2021), the main reason is that the process of spinal cord injury development is accompanied by excessive production of free radicals, among which oxidative stress causes the spinal cord to be susceptible to oxidative damage, thereby triggering oxidative stress (Zhang H, et al., 2024; Yao H, et al., 2024). At the same time, a large number of studies have shown that ncRNAs play an important regulatory role in oxidative stress, and the abnormal expression of ncRNAs causes the occurrence of oxidative stress (Table 1; Figure 1).

Overexpression of circZFHX3 (Tian et al., 2022), circWdfy3 (Shao et al., 2024a), lncTCTN2 (Liu et al., 2022), miR-340-5p (Qian et al., 2020) increases their expression levels by directly or indirectly acting on downstream targets, thereby enhancing cell viability, reducing ROS accumulation, reducing oxidative stress, and promoting the recovery of motor function. In addition, overexpression of lncOIP5-AS1 improves mitochondrial function and reduces oxidative stress through the miR-128-3p/Nrf2 axis, and the specific mechanism is that overexpression of lncOIP5-AS1 indirectly leads to an increase in Nrf2 levels by increasing the spongy effect on miR-128-3p, thereby improving mitochondrial function, reducing oxidative stress, and promoting the recovery of spinal cord injury (Jiang et al., 2024).

Oxidative stress induced by spinal cord injury is caused by ROS accumulation and lipid peroxidation on the one hand, and mitochondrial function impairment on the other hand (Cui et al., 2025). Therefore, activation of antioxidant pathway cannot completely solve the damage caused by oxidative stress. Meanwhile, mitochondrial function should be repaired. By regulating the genes related to mitochondrial dynamics controlled by exosomes, mitochondrial membrane potential and ATP synthesis should be improved to alleviate energy metabolism disorders. Exosomal RNA has regulatory effects in both aspects. Therefore, the effect of exosomal RNA treatment is better than antioxidant treatment alone.

## 5 NcRNA regulation of inflammatory response after spinal cord injury

Inflammation is considered an important pathological process in the secondary injury phase, which can directly or indirectly determine the therapeutic effect of spinal cord injury. Inflammatory response can greatly trigger a series of secondary injuries, leading to neuronal death and ultimately resulting in neurological dysfunction after injury (Lu et al., 2024). Numerous studies have shown that ncRNAs play a crucial role in regulating the inflammatory response, which may become an important means of treating and improving spinal cord injury (Table 1; Figure 1).

The mechanism of action of low-expression circGla is that circGla, as a competitive endogenous RNA of miR-488, indirectly reduces the expression of MEKK1 by acting as a sponge, thereby reducing the inflammatory state of astrocytes (Shao et al., 2024b). Overexpression of circZFHX3 activates microglia, promotes cell viability, and inhibits inflammatory responses (Tian et al., 2022). Overexpression of circHIPK3 can increase DUSP1 expression through the miR-382-5p/DUSP1 axis, thereby reducing the cellular inflammatory response (Yin et al., 2022). Low expression circPedia-4214 promote macrophage M2 polarization and participate in the immuno-inflammatory response (Cao et al., 2023). Overexpression of circWdfy3 reduces the accumulation of inflammatory factors and improves the prognosis after spinal cord injury (Shao et al., 2024a).

Low-expression lncZFAS1 indirectly inhibits PTEN expression by binding to miR-1953, thereby indirectly activating the PI3K/AKT pathway to further inhibit the inflammatory response, thereby promoting spinal cord function recovery after spinal cord injury (Chen et al., 2021). In addition, low expression of lncAirsci (Zhang T, et al., 2021), lncNEAT1 (An et al., 2021), lncGAS5 (Cao et al., 2021), lncXIST (Zhong et al., 2021) can reduce the inflammatory response, thereby promoting functional recovery after spinal cord injury. Overexpression of lncCCAT1 (Xia et al., 2020) and lncTCTN2 indirectly increases the expression level of downstream proteins through spongy action on downstream miRNAs, thereby reducing the inflammatory response and promoting functional recovery of spinal cord injury (Liu et al., 2022).

Overexpression of lncMEG3 (Zhou et al., 2022) and lncGm37494 (Shao et al., 2020) and low expression of lncXIST (Yang et al., 2023b) and lncTUG1 (Ju et al., 2023) inhibit M1-type polarization of microglia through indirect regulation of downstream targets, promote M2-type polarization, and secrete anti-inflammatory factors to reduce inflammatory response. In addition, overexpression of let-7b-5p attenuated pyroptosis in microglia/macrophages by inhibiting its downstream target gene LRIG3, thereby reducing the secondary inflammatory response after spinal cord injury (Liu et al., 2024).

Overexpression of miR-124-3p reduces neuroinflammation through the MYH9/PI3K/AKT/NF-κB signaling pathway, and the main mechanism is that overexpression of miR-124-3p can inhibit MYH9, and inhibition of the MYH9 signaling pathway can activate the PI3K/AKT signaling pathway and inhibit the NF-κB signaling pathway, which in turn inhibits A1 astrocytes, thereby inhibiting the activation of M1 microglia and microglia-induced neuroinflammatory response (Jiang et al., 2020). Overexpression of miR-340-5p can reduce the inflammatory response by enhancing the inhibition of the downstream target P38-MAPK signaling pathway (Qian et al., 2020).

It can be seen that inflammation is the dominant factor in the pathological mechanism of spinal cord injury, and ncRNAs play an important role in the regulation of inflammatory response, which can achieve indirect regulation of inflammatory response by regulating the expression of ncRNAs, thereby improving spinal cord injury. However, for inflammatory response, anti-inflammatory alone cannot completely solve the progression of the disorder. Studies have shown that inflammation-oxidative stress is intermodulated (Xiong et al., 2023), so that the best therapeutic effect can be achieved through a dual antioxidant-anti-inflammatory targeting strategy.

## 6 NcRNA regulation of autophagy after spinal cord injury

Autophagy is a lysosomal degradation pathway for cytoplasmic components and organelles, which is crucial for maintaining cellular homeostasis and defending against external stress (Yao H, et al., 2024; Li et al., 2022). Studies have found that autophagy plays an important role in improving spinal cord injury, as it alleviates nerve damage by regulating microtubule dynamics and mediating axonal regeneration, thereby exerting neuroprotective effects on spinal cord injury (Geng et al., 2024). Additionally, autophagy can inhibit systemic inflammatory responses, reduce tissue damage and neuronal cell death induced by inflammatory cascades, and promote the recovery of neurological function (Zhang H, et al., 2024). Through continuous exploration, it has been discovered that ncRNAs play an important role in the regulation of autophagy, and perhaps by indirectly regulating the expression of ncRNAs to achieve the regulation of autophagy, it can become a new treatment method for spinal cord injury (Table 2; Figure 1).

**Table 2:** Regulation of the pathogenesis of spinal cord injury by ncRNAs.

ncRNAs	Expression	RNA regulatory axis expression	SCI pathogenesis	References
NcRNA regulation of autophagy after spinal cord injury				
circAstn1	↑	circAstn1↑/ miR-138-5p↓/Atg7↑	autophagy ↑	Shao et al. (2025)
circHIPK2	↑	circHIPK2↑/ miR-124-3p↓/Smad2↑	autophagy ↑	Yang et al. (2024)
lncSNHG1	↓	lncSNHG1↓/ miR-362-3p↑/ Jak2/stat3↓	autophagy ↓	Zhou et al. (2021)
lncMALAT1	↑	lncMALAT1↑/ miR-22-3p↓/SIRT1/AMPK↑	autophagy ↑	Li et al. (2023)
NcRNA regulates cell apoptosis after spinal cord injury				
circZFHX3	↑	circZFHX3↑/ miR-16-5p↓/IGF-1↑	apoptosis ↓	Tian et al. (2022)
circHIPK3	↑	circHIPK3↑/ miR-382-5p↓/DUSP1↑	apoptosis ↓	Yin et al. (2022)
lncCCAT1	↑	lncCCAT1↑/ miR-218↓/NFAT5↑	apoptosis ↓	Xia et al. (2020)
lncMIAT	↑	lncMIAT↑/ RBFOX2↑	apoptosis ↓	He et al. (2022)
lncOIP5-AS1	↑	lncOIP5-AS1↑/ miR-128-3p↓/Nrf 2↑	apoptosis ↓	Jiang et al. (2024)
lncRMRP	↑	lncRMRP↑/ miR-766-5p↓/FAM83A↑	apoptosis ↓	Hong et al. (2022)
lncTSIX	↑	lncTSIX↑/ miR-532-3p↓/ DDOST↑	apoptosis ↓	Dong et al. (2023)
lncSNHG1	↓	lncSNHG1↓/ miR-362-3p↑/ Jak2/stat3↓	apoptosis ↓	Zhou et al. (2021)
lncGAS5	↓	lncGAS5↓/ miR-93↑/PTEN↓	apoptosis ↓	Cao et al. (2021)
lncTCTN2	↑	lncTCTN2↑/ miR-329-3p↓/IGF1R↑	apoptosis ↓	Liu et al. (2022)
lncRMRP	↑	lncRMRP↑/ EIF4A3↓/SIRT1↑	apoptosis ↓	Wang et al. (2025)
lncMALAT1	↑	lncMALAT1↑/ miR-22-3p↓/SIRT1/AMPK↑	apoptosis ↓	Li et al. (2023)
lncXIST	↓	lncXIST↓/ miR-219-5p↑/NF-κB↓	apoptosis ↓	Zhong et al. (2021)
lncZFAS1	↓	lncZFAS1↓/ miR-1953↑/ PTEN/ PI3K/AKT↓	apoptosis ↓	Chen et al. (2021)
miR-339	↓	miR-339↓/ PDXK↑	apoptosis ↓	Xiong et al. (2022)
miR-340-5p	↑	miR-340-5p↑/ P38/MAPK↓	apoptosis ↓	Qian et al. (2020)

High expression of circAstn1 activates autophagy through the miR-138-5p/Atg7 (Autophagy related 7) pathway to promote spinal cord repair after injury. Both miR-138-5p and Atg7 are downstream targets of circAstn1, and high expression of circAstn1 enhances its sponge effect on miR-138-5p, reducing its expression and indirectly increasing Atg7 expression (Shao et al., 2025). Overexpression of circHIPK2 promotes autophagy and endoplasmic reticulum (ER) stress through the miR-124-3p/Smad2 pathway, further enhancing the activation of A1 astrocytes after spinal cord injury (Yang et al., 2024).

Low expression of lncSNHG1 reduces the sponge effect on miR-362-3p, indirectly inactivating the Jak2/Stat3 pathway and reducing cell autophagy (Zhou et al., 2021). Overexpression of lncMALAT1 promotes the SIRT1 (Sirtuin 1) /AMPK (AMP-activated protein kinase) pathway through the miR-22-3p/SIRT1/AMPK axis, activating autophagy and thereby exerting neuroprotective effects, promoting the recovery of neurological function in spinal cord injury (Li et al., 2023).

Autophagy reduces inflammation and oxidative stress by clearing damaged organelles in the early stages of spinal cord injury, but excessive activation can exacerbate neuronal death (Shen et al., 2023), so regulation of autophagy may become an important means to improve spinal cord injury in the future. Exosomes encapsulate ncRNAs (acting as autophagy inhibitors) and target them to the injury site, inhibiting neurodegeneration caused by excessive autophagy, thus achieving motor neural function recovery after spinal cord injury.

## 7 NcRNA regulates cell apoptosis after spinal cord injury

After spinal cord injury, the adverse microenvironment of the injury, such as ischemia and hypoxia, free radical release, and acute inflammation, leads to the death of neuronal cells (Ji et al., 2024), among which apoptosis is widely considered to be the key to secondary injury, which is the main reason for the deterioration of neurological function after spinal cord injury other than the primary mechanical injury (Yao et al., 2020). In addition, a large number of studies have found that in central nervous system disorders, inhibiting neuronal apoptosis will be beneficial to the recovery of motor function after spinal cord injury (Yin et al., 2022). At the same time, more and more evidence shows that ncRNAs are closely related to cell proliferation and apoptosis after spinal cord injury. Perhaps by regulating the expression of ncRNAs (Table 2; Figure 1), indirect regulation of cell proliferation and apoptosis can be achieved, which can become a new method for treating and improving spinal cord injury.

Overexpression of lncMALAT 1 promotes the SIRT1/AMPK pathway through the miR-22-3p/SIRT1/AMPK axis, inhibits apoptosis in nerve cells, and then exerts a neuroprotective role and promotes the recovery of nerve function after spinal cord injury (Li et al., 2023). In addition, overexpression of circZFHX3 (Tian et al., 2022), circHIPK3 (Yin et al., 2022), lncCCAT1 (Xia et al., 2020), lncMIAT (He et al., 2022), lncOIP5-AS1 (Jiang et al., 2024), lncRMRP (Hong et al., 2022; Wang et al., 2025), lncTSIX (Dong et al., 2023) and lnc TCTN2 (Liu et al., 2022) indirectly increased the expression of downstream proteins, thereby reducing apoptosis and promoting the recovery of motor function. Overexpression of miR-340-5p reduced apoptosis by inhibiting the P38/MAPK pathway, thereby promoting the recovery of neurological function after spinal cord injury (Qian et al., 2020).

Low expression of lncSNHG1 (Zhou et al., 2021), lncGAS5 (Cao et al., 2021), lncXIST (Zhong et al., 2021), and lncZFAS1 (Chen et al., 2021) inhibits apoptosis by inhibiting downstream proteins, thereby promoting spinal cord function recovery after spinal cord injury. Low expression of miR-339 can target PDXK, and PDXK overexpression can significantly improve motor function, increase neuronal activity, reduce neuronal apoptosis, and improve spinal cord injury (Xiong et al., 2022).

In recent years, significant progress has been made in the research on apoptosis caused by exosomal RNA regulation of spinal cord injury, the core of which lies in the regulation of apoptosis-related pathways by non-coding RNAs to inhibit secondary damage and promote nerve repair. At the same time, cell proliferation should also be promoted through exosomal RNA regulation. It can reduce apoptosis and promote cell proliferation, and achieve a better and more effective treatment strategy by inhibiting apoptosis and promoting proliferation.

## 8 Prospect

Spinal cord injury is a severe central nervous system disorder with complex pathogenesis that often leads to significant disability. However, despite advances in surgical techniques, there is still no effective treatment for this debilitating condition (Karsy and Hawryluk, 2019; Liu et al., 2025). After spinal cord injury, the blood-spinal cord barrier is disrupted, leading to immune microenvironmental disturbances and poor regeneration of the injured spinal cord (Valido et al., 2023; Al Mamun et al., 2021). Due to the decline in immune cell function, spinal cord injury patients exhibit a higher incidence of infections. Individuals experience a transition from the acute to the chronic phase, during which changes in gene expression are also time-dependent (Mun et al., 2022). Therefore, further exploration of the molecular mechanisms and changes in the microenvironment after spinal cord injury is crucial for developing better treatment strategies.

The blood-spinal cord barrier (BSCB), conceptually equivalent to the blood–brain barrier (BBB) in the spinal cord, provides a functional microenvironment similar to that of the BBB for spinal cord cellular components; therefore, the BSCB is considered a morphological extension of the BBB (Bartanusz et al., 2011), spinal cord injury also causes direct vascular damage and significant disruption of the BSCB (Sun et al., 2022; Whetstone et al., 2003). BBB damage has become an important factor in determining the progression and prognosis of central nervous system disorders, but currently, there are no clinical pharmacological treatments that directly address BBB dysfunction (Ihezie et al., 2021). Over the past decade, the involvement and regulatory functions of non-coding RNAs in BBB dysfunction in CNS disorders have been rapidly and extensively studied. A large body of evidence has demonstrated the effectiveness and capacity of miRNAs, lncRNAs, and circRNAs in protecting the BSCB in conditions such as spinal cord injury (Li et al., 2021).

Recent studies have shown that cell therapy plays an important role in the treatment of spinal cord injury. However, the therapeutic effects of cell transplantation in spinal cord injury models are still controversial, and their clinical application is limited by several factors, including potential tumorigenic risks (Zhang M, et al., 2024) and ethical concerns (Margiana et al., 2022). However, research indicates that exosomes derived from stem cells have anti-inflammatory effects and play an irreplaceable role in the treatment of spinal cord injury. As a new type of regenerative medicine therapeutic, they have advantages such as small size, low immunogenicity, and the ability to cross the blood-spinal cord barrier (Zhang et al., 2023).

However, despite the great potential of exosomal ncRNAs, its application as a therapeutic agent still faces significant challenges. One major obstacle is the effective monitoring and guidance of exosomes to reach their target receptor areas. Exosomes are small vesicles released by cells, making them difficult to track and control once administered (Guo et al., 2025; Wang et al., 2022), and there are also issues such as short duration of action. However, biomaterials are of great value in treating and repairing damaged tissues, as well as in assisting drug delivery and release. Emerging biomaterials not only aim to restore the structure and function of damaged tissues but also promote their regeneration through active and targeted interactions.

Compared with traditional drug interventions and surgical treatments, the use of biomaterial scaffolds can reduce some of the complex side effects of drugs and obstacles to functional recovery after surgery. However, biomaterials may be recognized as foreign objects by the patient’s immune system, triggering inflammatory reactions. This reaction may exacerbate the inflammatory microenvironment after spinal cord injury, further damaging neural tissue. Therefore, it is crucial to develop a treatment method that can target controlled drug delivery with minimal side effects.

Exosomes have strong biological activity but suffer from issues such as short duration of action, while biomaterials (such as hydrogels and scaffolds) can serve as sustained-release carriers for exosomes, prolonging their retention time at the injury site and improving the therapeutic effect (Yang et al., 2023a). The combination of the two can compensate for their respective shortcomings and improve the therapeutic effect. Perhaps in the future, through material engineering and gene editing techniques, the combination of exosomes and biomaterials can be further optimized, such as designing materials with specific degradation rates to match the release kinetics of exosomes, thereby improving the utilization efficiency of exosomes. At the same time, this can also reduce costs and be more conducive to clinical translation. On the premise of verifying the safety and effectiveness of the combination therapy, personalized biomaterial and exosome combination schemes can be designed based on the patient’s specific condition, which is expected to achieve more precise personalized treatment.

## 9 Conclusion

In recent years, the understanding of the pathological mechanisms of spinal cord injury has deepened, and at the same time, it has been discovered that the abnormal expression of ncRNA plays an increasingly important role in the regulation of the pathological mechanisms of spinal cord injury. ncRNA plays an important role in the pathophysiological process after spinal cord injury, including synaptic regeneration, oxidative stress, inflammatory response, autophagy, and cell proliferation and apoptosis. These processes are interwoven and collectively influence the outcome of spinal cord injury. Based on the critical role of ncRNA in the pathophysiology of spinal cord injury, ncRNA can be regarded as a potential therapeutic target. By regulating ncRNA, these processes can be intervened, providing new strategies for the treatment of spinal cord injury. For example, by designing specific ncRNA mimics or inhibitors, the expression and function of ncRNA can be targeted for regulation, thereby achieving targeted treatment of spinal cord injury.
